# Comprehensive Analysis of a Platelet- and Coagulation-Related Prognostic Gene Signature Identifies CYP19A1 as a Key Tumorigenic Driver of Colorectal Cancer

**DOI:** 10.3390/biomedicines12102225

**Published:** 2024-09-30

**Authors:** Guoqing Su, Meiqin Wang, Jinghang Qian, Yang Wang, Yu Zhu, Nannan Wang, Ke Wang, Qifan Wang, Yi Wang, Dongzheng Li, Liu Yang

**Affiliations:** Department of Colorectal Surgery, The Affiliated Cancer Hospital of Nanjing Medical University & Jiangsu Cancer Hospital & Jiangsu Institute of Cancer Research, Nanjing 210009, China; guoqingsunjmu@stu.njmu.edu.cn (G.S.); meiqin-wang@163.com (M.W.); q1222jh@163.com (J.Q.); 18956308270@163.com (Y.W.); 13245822976@163.com (Y.Z.); nannanwang@163.com (N.W.); kewang@163.com (K.W.); qifanwang@163.com (Q.W.); wy2023@stu.nimu.edu.cn (Y.W.)

**Keywords:** colorectal cancer, coagulation, platelet, CYP19A1, weighted gene co-expression network analysis, single-cell sequencing, survival, prognosis

## Abstract

Background: The intricate interplay between the platelet-coagulation system and the progression of malignant tumors has profound therapeutic implications. However, a thorough examination of platelet and coagulation markers specific to colorectal cancer (CRC) is conspicuously absent in the current literature. Consequently, there is an urgent need for further exploration into the mechanistic underpinnings of these markers and their potential clinical applications. Methods: By integrating RNA-seq data and clinicopathological information from patients with CRC in the cancer genome atlas, we identified genes related to the platelet-coagulation system using weighted gene co-expression networks and univariate Cox analysis. We established a prognostic risk model based on platelet- and coagulation-related genes using Lasso Cox regression analysis and validated the model in two independent CRC cohorts. We explored potential biological functional disparities between high-risk and low-risk groups through comprehensive bioinformatics analysis. Results: Our findings indicate that colorectal cancer patients classified as high-risk generally exhibit poorer prognoses. Moreover, the model’s risk scores were associated with the differential composition of the immune tumor microenvironment, suggesting its applicability to infer immunotherapy responsiveness. Cellular functional experiments and animal experiments indicated that CYP19A1 expression in CRC influences malignant phenotype and platelet activation. Conclusions: In summary, we present a novel platelet- and coagulation-related risk model for prognostic assessment of patients with CRC and confirm the important role of CYP19A1 in promoting malignant progression of CRC.

## 1. Introduction

Colorectal cancer (CRC) ranks among the most prevalent malignancies of the gastrointestinal tract [1,2,3]. It is the third most frequently diagnosed cancer worldwide and has become a leading cause of cancer-related mortality [4,5]. The 5-year survival rates for CRC show notable variation across different studies, ranging from 91% to 80% for histologic stages I and II, and decreasing to 61.7% and 23.2% for histologic stages III and IV [6,7]. Alarmingly, about 42% of patients experience local recurrence or distant metastases during stages II and III [8]. Remarkably, cancer-associated thrombosis often serves as an initial harbinger of malignancy and is currently the second leading cause of mortality, following the cancer itself, among patients with cancer [9]. In numerous malignancies, platelet activation and coagulation are intricately intertwined with malignant tumor progression and often predict an unfavorable prognosis for patients [9,10]. Consequently, there is a pressing need for the development of platelet- and coagulation-related prognostic markers to stratify risk among patients with CRC and enable timely interventions.

A growing body of evidence highlights the pivotal role of tumor-associated platelets in driving cancer progression [11,12]. Platelets, as multifaceted actors in tumorigenesis and the largest and most widely distributed circulating reservoir of tumor-promoting factors within the human body, have emerged as valuable biomarkers and diagnostic tools across various cancer types [12]. Of particular note, platelet-derived transforming growth factor-β (TGF-β) and direct platelet–tumor cell interactions synergistically promote epithelial–mesenchymal transition (EMT) in tumor cells, thereby fostering metastasis [13]. Furthermore, platelet aggregation and degranulation, coupled with the subsequent release of platelet-derived proangiogenic mediators, are instrumental in shaping the TME and impacting tumor growth [14]. Most notably, thromboembolism resulting from thrombocytosis plays a pivotal role in cancer progression and is intimately linked with adverse survival prognosis in patients with cancer [15].

CRC stands as a prevalent malignancy, with platelets assuming a pivotal role in its tumorigenesis and metastatic cascade [15]. Within the tumor microenvironment (TME), tumor-associated platelets emerge as a dynamic force, engaging in intricate interactions with tumor cells, thereby fostering tumor proliferation, progression, and metastasis [16]. Notably, thrombocytosis serves as a marker for heightened susceptibility to cancer-related thrombosis [17]. It has been shown that elevated platelet levels and the presence of tumor-derived extracellular vesicles collaborate in promoting thrombosis in cancer-afflicted individuals [18,19]. Approximately 20% of patients with cancer suffer from vascular thromboembolism, with an escalated thrombotic risk particularly discernible in those diagnosed with brain, pancreatic, colorectal, and hematological cancers [20,21,22]. Hence, the inhibition of platelet activation and the ensuing coagulation cascade has emerged as an enticing avenue of research in cancer therapy.

We obtained datasets from various publicly available databases and conducted a comprehensive array of bioinformatics analyses. Initially, we employed Weighted gene co-expression network analysis (WGCNA) to identify a gene set closely related to the platelet-coagulation system. Subsequently, we undertook a one-way Cox-LASSO-multifactorial Cox regression approach to construct a prognostic model linked to a platelet- and coagulation-related gene (PCRG) set and further developed an interactive nomogram for dynamic risk assessment. To examine the correspondence of the risk model with the tumor’s immune status, analyses were conducted using gene set variation analysis (GSVA), gene set enrichment analysis (GSEA), and the CIBERSORT algorithm. Furthermore, the interplay of our prognostic model with the TME was scrutinized by establishing associations at the single-cell level. In our concluding analysis, we explored the link between the expression of CYP19A1, which emerged as a notable PCRG marker, and CRC progression. The present results highlight CYP19A1 as a novel prognostic biomarker for CRC, stress its pivotal pro-tumorigenic role, and reveal it as a promising target for therapeutic intervention.

## 2. Materials and Methods

### 2.1. Data Collection and Collation

RNA-seq data and clinical information for TCGA-COAD (colon adenocarcinoma), comprising 41 normal tissue samples and 469 CRC samples, were sourced from the TCGA database (https://portal.gdc.cancer.gov/ (accessed on 22 May 2024)). Samples with incomplete clinical and survival data were excluded to finally construct a training set encompassing 453 patients with COAD [23]. For exhaustive analysis, we retrieved the COAD probe matrix files (GSE39582_series_matrix and GSE17536_series_matrix) and the platform file (GPL 570-55999) from the GEO database [24]. To enhance data clarity, we acquired corresponding platform annotation files, converted probes to gene symbols, and computed the average expression levels of multiple probes corresponding to the same gene symbol, ensuring robust gene expression quantification. To establish a foundation for our investigation, we gathered coagulation-related genes from the GSEA website [25]. Concurrently, we extracted platelet-associated genes from the GeneCard website and meticulously screened for mRNAs exhibiting correlation coefficients > 1 [26]. Furthermore, we leveraged single-cell data from GSE221575, obtained from the GEO database. Of note, all included samples pertained to newly diagnosed patients. To ensure data accuracy and comparability, we rigorously normalized the raw count data using the “voom” function within the “limma” package in R (version 4.2.2) [26].

### 2.2. Statistical Analysis

Data analysis was conducted using R version 4.2.2.A *p*-value of less than 0.05 was considered statistically significant. In the figures, significance levels are marked as follows: * *p* < 0.05, ** *p* < 0.01, *** *p* < 0.001, and **** *p* < 0.0001.

### 2.3. Coagulation Co-Expression Network Construction and Differential Analysis

We harnessed the GSVA package (v1.44.1) within the R environment to compute enrichment scores for each coagulation-related gene set within every CRC sample, employing the single sample gene set enrichment analysis (ssGSEA) method [27,28]. To construct a co-expression mRNA network specific to TCGA-COAD, we employed the WGCNA package. An optimal soft threshold (β) was systematically determined to meet the requisite criteria for a scale-free network. Subsequently, the weighted adjacency matrix underwent transformation into a topological overlap matrix (TOM), along with the corresponding dissimilarity matrix (1-TOM). For module identification, we adopted a dynamic tree-cutting method. Specifically, we sought mRNA modules that exhibited statistically significant correlations with the coagulation enrichment score. Among those, the module demonstrating the highest correlation (herein referred to as the “coagulation module”) was selected for further investigation. Subsequently, we conducted an analysis of the differentially expressed coagulation module genes between the normal and tumor groups in the TCGA database. We utilized the “DESeq2” package with stringent criteria (*p*-value < 0.05 and |log FC| > log_2_(1.2)), and visually represented the results through volcano plots. Finally, we performed an intersection analysis of the platelet-related gene set with the differentially expressed coagulation module genes, thereby deriving a set of PCRGs that served as the foundation for our ensuing analyses.

### 2.4. Construction of a Prognostic Model and Survival Analysis

Utilizing data from the TCGA discovery cohort, we conducted univariate Cox proportional hazards regression analyses to identify PCRGs considerably associated with CRC overall survival (OS). Next, the most suitable PCRGs were identified using the least absolute shrinkage and selection operator (LASSO) regression method. To derive a risk score for each patient, we computed the score for each PCRG by multiplying its expression levels with the corresponding coefficients. These scores were then summed, resulting in a personalized risk score for each patient. To stratify patients in the discovery group, we employed the median risk score as a threshold, categorizing them into high-risk and low-risk groups. Kaplan–Meier survival curves and ROC analyses were performed for the discovery cohort.

Similarly, we extended our analysis to two validation cohorts, GSE39582 and GSE17536. As described earlier, these cohorts were similarly stratified into high-risk and low-risk groups. This categorization, combined with survival prognosis data, served as the validation phase to assess the precision and reliability of our prognostic model.

### 2.5. Nomogram Model Construction

The nomogram model for the TCGA cohort was established incorporating risk scores along with clinicopathologic variables encompassing age, gender, TNM stage, and race. To assess the model’s performance, calibration curves were employed to gauge the concordance between predicted and observed outcomes.

### 2.6. GSVA and GSEA

GSVA and GSEA analyses (performed in R version 4.2.2) were employed to explore the differences in biological functions. Additionally, we performed correlation analyses between risk scores and the expression levels of these terms, focusing on those with *p*-values < 0.05 and strong correlation coefficients [28].

### 2.7. Immune Cell Infiltration Analysis

The CIBERSORT algorithm was used to investigate the relationship between immune cell infiltration and projected risk values [29].

### 2.8. Single-Cell RNA Sequencing

Subsequently, we processed the single-cell expression data matrix employing the “Seurat” R package [30]. Initially, data from the GSE221575 dataset underwent normalization utilizing the “Normalize Data” function. Following this, we employed the “Find Variable Genes’ function to identify a set of 2000 highly variable genes. To augment the gene diversity and enhance gene expression correlation, we utilized pseudo-cells [31,32]. Next, we harnessed the “Find Integration Anchors’ and “Integrate Data” functions to integrate data from six distinct CRC samples. Upon completing the “Run PCA” function, we constructed a K-nearest neighbor graph based on Principal Component Analysis (PCA) using the “Find Neighbors” function. Subsequently, we employed the “Find Clusters” function to iteratively amalgamate cells, aiming for optimal resolution. Finally, we employed Uniform Manifold Approximation and Projection (UMAP) for visualization.

### 2.9. Cell Culture

We sourced the CRC cell lines HT29 and SW480, along with the human embryonic intestinal mucosa cell line CCC-HIE-2, from the American Type Culture Collection (ATCC, Manassas, VA, USA). SW480 and CCC-HIE-2 cells were cultured in Dulbecco’s Modified Eagle Medium (DMEM, Gibco, Shanghai, China), whereas McCoy’s 5A medium (Gibco, Shanghai, China) was used for HT29 cells. The culture media were further enriched with 10% Fetal Bovine Serum (FBS, Gibco, Shanghai, China) and 1% Streptomycin/Penicillin (Gibco, Shanghai, China). Cell incubation conditions were maintained at a humidified atmosphere of 37 °C with 5% CO_2_.

### 2.10. Transfection

To establish the stable CYP19A1-knockdown CRC cell lines, the human lentivirus-sh-CYP19A1 were bought from Corues Biotechnology (Nanjing, China). Small interfering RNA (siRNA) sequences targeting CYP19A1 (1-GCGCAAAGCCUUAGAAGAUTT, 2-GAGAAAGGCAUCAUAUUUATT) and a non-targeting negative control siRNA (UUCUCCGAACGUGUCACGUTT) were procured from Corues Biotechnology (Nanjing, China). The transfection was carried out following the Lipofectamine 3000 protocol, and cell lines were selected using puromycin.

### 2.11. Real Time Quantitative PCR (RT-qPCR)

Following the manufacturer’s guidelines, RNA was isolated using TRIzol reagent (Vazyme, Nanjing, China). cDNA synthesis was performed with ABScript III RT Master Mix (ABclonal, Wuhan, China), and quantitative PCR was conducted using Universal SYBR Green Fast qPCR Mix (ABclonal) according to the Bio-Rad detection system protocol (Bio-Rad, Berkeley, CA, USA). Beta-Actin (ACTB) was used as a reference gene, and the 2^−∆∆Ct^ method was employed to quantify target mRNA levels. The primers used in this research (listed in Supplementary File S7) were synthesized by Sangon Biotech (Shanghai, China).

### 2.12. Western Blotting

HT29 and SW480 CRC cells were lysed in RIPA buffer supplemented with protease and phosphatase inhibitors, then centrifuged at 12,000 rpm for 15 min at 4 °C. The resulting supernatant was combined with 5× loading buffer, heated at 95 °C for 10 min, and subjected to SDS-PAGE. Proteins were transferred onto a 0.45 μm PVDF membrane, blocked with 5% non-fat milk, and incubated with an anti-CYP19A1 primary antibody (1:1000, AS014, Abclonal) at 4 °C for 16 h. The membrane was then treated with a secondary antibody at room temperature for 2 h. Protein bands were visualized using a chemiluminescence imaging system and analyzed with Image Lab software (Bio-Rad, Hercules, CA, USA). GAPDH mouse monoclonal antibody (1:20,000, AC033, Abclonal) served as an internal reference for comparing the different treatment groups.

### 2.13. Colony Formation Assay

HT29 and SW480 cells were trypsinized, counted, and seeded at 500 cells per well in 6-well plates. The cells were cultured at 37 °C in a 5% CO_2_ environment for 14 days, with the medium changed every three days. After incubation, cells were fixed with 4% paraformaldehyde, stained with 0.5% crystal violet, and rinsed. Colonies containing more than 50 cells were counted under a microscope to assess colony-forming efficiency.

### 2.14. Cell Proliferation Assay

The 5-ethynyl-2′-deoxyuridine (EdU) incorporation assay was employed to assess cell proliferation. HT29 and SW480 cells were exposed to 10 μM EdU and incubated at 37 °C for 2 h. After incubation, cells were fixed with 4% paraformaldehyde, permeabilized, and subjected to a click chemistry reaction with an azide-fluorochrome conjugate. DAPI was used for nuclear staining. The proliferation rate was determined by visualizing and quantifying EdU-positive cells using fluorescence microscopy (CarlZeiss, Oberkochen, Germany).

### 2.15. Apoptosis and Necrosis Assay

Apoptosis and necrosis in HT29 and SW480 cells were evaluated using dual staining. Cells were mixed with 5 μL Annexin V-FITC (BD Biosciences, Franklin Lakes, NJ, USA) and 5 μL propidium iodide (PI), then incubated in the dark at room temperature for 20 min to avoid photobleaching. After incubation, cells were washed twice with phosphate-buffered saline (PBS) and resuspended in 500 μL PBS. A minimum of 20,000 cells per sample were analyzed using a CytoFlex flow cytometer (Beckman Coulter, Brea, CA, USA), and apoptotic and necrotic populations were quantified with FlowJo software (FlowJo v10).

### 2.16. Cell Cycle Analysis

HT29 and SW480 cells were washed in PBS and then incubated for 30 min in a staining solution containing 500 μg/mL PI and 100 μg/mL RNase A. This process facilitates the intercalation of PI into the DNA, enabling the assessment of cellular DNA content. Post-incubation, the cells were subjected to flow cytometric analysis for quantitative evaluation of DNA contents and cell cycle status.

### 2.17. Transwell Assay

HT29 and SW480 cells were seeded in the upper chambers of Transwell inserts (Millicell, Darmstadt, Germany; 8 µm pore size) coated with Matrigel, while 500 µL of complete medium was added to the lower wells. After incubating for 72 h at 37 °C, non-migrating cells in the upper chambers were carefully removed with a cotton swab. Cells that migrated to the lower chambers were fixed for 10 min and stained with 1% crystal violet in 2% ethanol for 15 min. This process enabled the quantitative assessment of cell migration.

### 2.18. Wound-Healing Assay

Once HT29 and SW480 cells reached 90% confluence, a linear scratch was created on the cell monolayer with a sterile pipette tip. Microscopic images of the scratch were captured immediately (0 h) and again 24 h later. Wound closure was assessed by measuring the average wound distance using ImageJ software (version 1.50i) to evaluate cell migration.

### 2.19. Platelet Activation Analysis

CYP19A1-silenced and control HT29 and SW480 cells were seeded into 24-well plates at 80% confluency. After the cells adhered, platelets from healthy human donors were added and incubated in direct contact with the CRC cells for 60 min. The platelets were then collected for flow cytometry analysis. Platelets were detected using the CD61 antibody, and their activation was assessed by labeling with the platelet surface markers P-selectin (CD62P) and CD41/CD61 (PAC-1). All experiments were performed independently at least three times. The primary antibodies used were FITC anti-human CD61 (336404; BioLegend, San Diego, CA, USA), PE anti-human CD62P (304906; BioLegend), and Alexa Fluor^®^ 647 anti-human CD41/CD61 (362806; BioLegend).

### 2.20. Animal Experiments

Four-week-old male BALB/c athymic nude mice were purchased from WeiTongHuaLi (Beijing, China) and housed according to the guidelines set by the Institutional Animal Care and Use Committee of Nanjing Medical University (Approval No. IACUC-2211031). We subcutaneously injected 2 × 10⁶ CYP19A1-knockdown or control HT29 and SW480 cells, suspended in 100 μL of cold PBS, into the dorsal flanks of the mice. Tumor volume was measured bi-weekly, calculated as V (mm^3^) = 1/2 × (length × width^2^), once the tumors became palpable. Upon study completion, the mice were humanely euthanized, and tumors were excised, individually weighed, and photographed.

To evaluate metastasis in vivo, we utilized a tail vein injection lung metastasis model. Luciferase-labeled cells, with 1 × 10⁶ cells in 100 μL of PBS, were injected into the tail vein of the mice. After five weeks, the mice were anesthetized with 2% isoflurane and injected with D-luciferin sodium salt (150 mg/mL) to detect metastatic lung lesions.

To further our investigation, we established a liver metastasis model via splenic injection. For this, a single-cell suspension of 1 × 10⁶ Lewis lung carcinoma cells expressing luciferase in 200 μL was prepared. Male nude mice (6–8 weeks old, 16–22 g) were anesthetized, and a 2–3 cm subcostal incision was made on the left side in the supine position. The spleen was exposed and ligated with 4-0 sutures, then divided between the ligatures to create two hemispans. Using a 27G syringe (BD, New Jersey(NJ),USA), 100 μL of the cell suspension was injected into one hemispan. After approximately 10 min, during which the cells were allowed to metastasize to the liver, the injected hemispan was excised. The remaining hemispan was returned to its original position in the abdominal cavity, and the incision was closed. Bioluminescence imaging was conducted on Days 0, 6, 10, and 14 post-injection to monitor tumor metastasis and liver growth, following a 3-minute luciferase substrate administration.

Bioluminescence was captured using the IVIS Spectrum imaging system (Caliper Life Sciences, Hopkinton, MA, USA). After imaging, the mice were euthanized, and the lungs and livers were collected for H&E staining. All procedures were performed in compliance with the guidelines of the Nanjing Medical University Committee for the Use and Care of Laboratory Animals, with ethical approval obtained.

## 3. Results

### 3.1. Identification of mRNA Modules Associated with Coagulation

**Figure 1:**
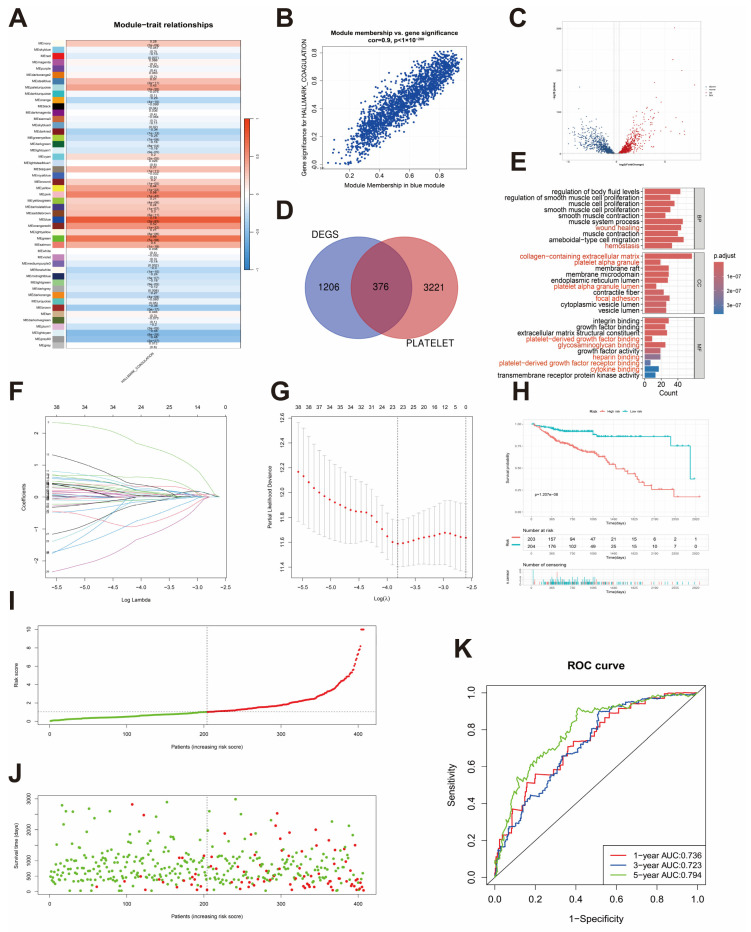
Construction of a platelet-coagulation-related prognostic model for CRC. (**A**) Heatmap of module–trait relationships. The blue module was considerably associated with the HALLMARK_COAGULATION gene set. (**B**) Scatter plot showing the correlation between gene module membership in the blue module and gene significance. (**C**) Differential analysis of blue module genes. (**D**) Venn diagram illustrating the overlap between differentially expressed genes (DEGs) in the blue module and the platelet-related gene set. (**E**) GO analysis of 376 platelet-coagulation-related genes (PCRGs) (The pathways labeled in red font are GO terms associated with platelet function). (**F**) Coefficient values of PCRGs. (**G**) Partial likelihood deviance of PCRGs. (**H**) Risk score and survival probabilities in the TCGA training set. (**I**,**J**) Distribution of risk scores and survival status in the TCGA-COAD cohort. (**K**) ROC curves for TCGA-COAD cases at 1, 3, and 5 years.

We first conducted single sample GSEA (ssGSEA) [33] to assess enrichment of the HALLMARK_COAGULATION gene set (MSigDB database; http://www.broad.mit.edu/gsea/msigdb/ (accessed on 22 May 2024)) within 453 CRC samples from TCGA. In the context of weighted gene co-expression network analysis (WGCNA), we determined that a soft threshold value (β) of 0.85 was optimal for constructing a robust co-expression network (Supplementary Figure S1A). Subsequently, we identified a total of 49 modules, each delineated by distinct color-coded sections within the graphical representation (Supplementary Figure S1B). Within each module, we defined intrinsic genes as the first principal component of gene expression. A heatmap provided insight into the interrelatedness of these genes within the modules (Supplementary Figure S1C). Furthermore, we computed correlations between the identified modules and the enrichment scores for the HALLMARK_COAGULATION pathway, and the strongest correlation was observed for the blue module (Figure 1A). The blue gene module exhibits a strong correlation (r = 0.9) between gene significance and module membership (MM), suggesting a high level of gene concordance within the module. (Figure 1B).

### 3.2. Differential Gene Expression Analysis and Identification of Coagulation-Associated Gene Sets

In total, we extracted 2046 coagulation-related genes from the blue module (Supplementary File S1). Subsequently, we used count data from the TCGA-COAD database to identify blue module genes that were differentially expressed between tumor and paracancerous (normal) samples. Specifically, we utilized the “DESeq2” package (version 1.38.3) to select genes that met the strict thresholds of |log_2_FC| > log_2_(1.2) and *p* < 0.05. Our analysis showed that 1582 genes were differentially expressed in tumor tissues compared to paracancerous tissues in the training set. Of these, 833 genes were upregulated in paracancerous tissues, whereas the remaining 749 genes showed elevated expression in COAD tissues (Figure 1C).

We retrieved a total of 1127 genes associated with platelets from the GeneCards database, employing specific screening criteria that included a correlation score > 1 and classification as protein-coding genes (Supplementary File S2). Subsequently, we conducted GO (Gene Ontology) and KEGG (Kyoto Encyclopedia of Genes and Genomes) analyses to elucidate the functions of these platelet-related genes. These analyses unveiled that the genes associated with platelets were prominently enriched in BP associated with coagulation, platelet alpha granules, and platelet activation, as illustrated in Supplementary Figure S1D,E. We next performed an intersection analysis between the aforementioned set of differentially expressed, coagulation-related module genes and the set of platelet-related genes, yielding a set of 376 genes that we termed PCRGs (Figure 1D). This PCRG set was subsequently utilized for further analyses. As expected, GO analysis of these genes revealed notable enrichment in BP associated with platelet function and coagulation (Figure 1E).

### 3.3. Development of a Platelet- and Coagulation-Related Risk Signature

Using the above subset of 376 PCRGs, we conducted univariate Cox regression analysis (*p* < 0.05) to identify among them those with prognostic relevance. This analysis led to the identification of 49 PCRGs (Supplementary File S3) that were subsequently utilized to construct a risk model through Lasso Cox regression analysis (Figure 1F,G). Subsequently, a risk profile comprising 10 selected PCRGs with corresponding expression levels and regression coefficients was formulated as: Risk Score = (2.790 * CYP19A1) + (0.419 * S1PR3) + (0.429 * COX4I2) + (1.223 * GPR162) + (−0.331 * F2RL2) + (−2.663 * SLC18A2) + (0.810 * MECP2) + (0.221 * LTBP1) + (−1.743 * HDC) + (−1.134 * HLF) (Supplementary File S4). Patients in the training cohort were stratified into high-risk (*n* = 203) and low-risk (*n* = 204) groups according to the median risk score. Kaplan–Meier (KM) survival curves demonstrated that high-risk patients exhibited worse OS compared to their low-risk counterparts (Figure 1H). Moreover, marked differences were observed in risk scores, survival outcomes, and gene expression profiles between the high-risk and low-risk groups. (Figure 1I,J and Supplementary Figure S1F). In turn, the area under the curve (AUC) in time-dependent ROC curves was evaluated at 1, 3, and 5 years, yielding values of 0.736, 0.723, and 0.794, respectively (Figure 1K).

### 3.4. External Validation of the Risk Assessment Model

**Figure 2:**
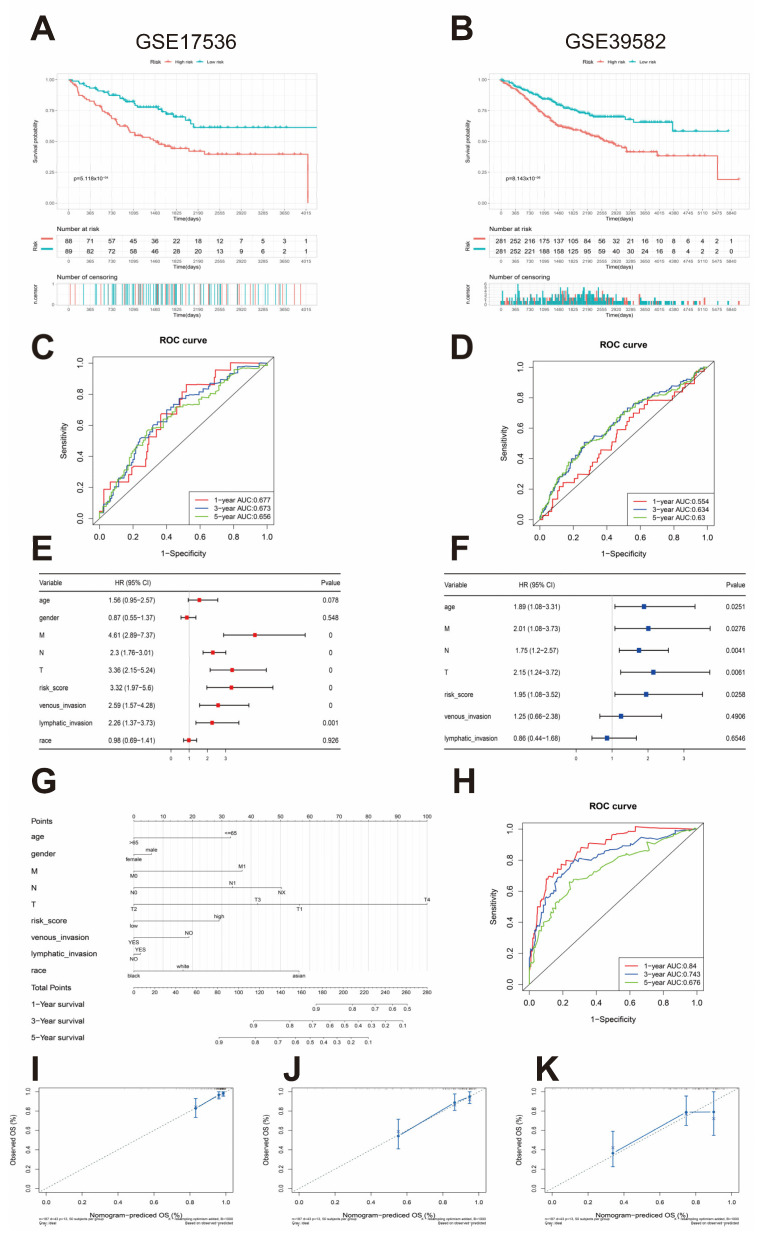
Validation of the prognostic model and development of a survival prediction nomogram. (**A**,**B**) Overall survival (OS) curves for patients in various risk groups based on the models, shown for the GSE17536 (**left**) and GSE39582 (**right**) cohorts. (**C**,**D**) ROC curves for predicting CRC patient outcomes at 1, 3, and 5 years in the GSE17536 (**left**) and GSE39582 (**right**) cohorts. (**E**,**F**) COX regression analysis for assessing the independent prognostic value of risk scores in the TCGA-COAD cohort. (**E**) Univariate regression analysis. (**F**) Multivariate regression analysis. (**G**) A nomogram integrating the risk score derived from PCRGs with clinicopathological factors was created to estimate 1-, 3-, and 5-year survival. (**H**) ROC curves were used to assess the nomogram’s prognostic accuracy. (**I**–**K**) Calibration curves showing the predictions of the nomogram for 1- (**I**), 3- (**J**), and 5-year (**K**) OS.

The predictive performance of our prognostic model was validated in two independent GEO cohorts, GSE39582 and GSE17536, using the same formula and optimal cutoff values. As illustrated in Figure 2A,B, both external datasets consistently demonstrated considerably worse OS among high-risk, compared to low-risk, patients. A visual representation of the disparities in risk score distribution, survival status, and expression patterns of risk genes between the two risk groups within these cohorts is provided in Supplementary Figure S2A–F. We evaluated the prognostic predictive capability of the risk model by analyzing the AUC values of the ROC curve. The results yielded annual standardized AUCs of 0.677, 0.673, and 0.656 in the GSE17536 cohort (Figure 2C) and of 0.554, 0.634, and 0.63 in the GSE39582 cohort (Figure 2D). These findings underscore the accuracy and robust stability of our risk model for prognostic assessment.

### 3.5. Nomogram Construction and Validation and Independent Prognostic Factor Analysis

Univariate Cox regression analysis confirmed a significant correlation between the risk model score and survival outcomes (Figure 2E). Moreover, the findings from multivariate Cox regression analysis proved that risk scores remained considerably correlated with survival even after adjusting for pertinent biological factors (Figure 2F). These observations collectively underscore the excellent predictive utility of our risk models for clinical applications. Subsequently, we crafted a clinical column-line graph integrating PCRGs with clinicopathologic features, which depicted 1-, 3-, and 5-year survival rates in patients with CRC (Figure 2G). This graphical representation evidences prognostic accuracy for short-term and long-term survival predictions. Additionally, we assessed the accuracy of our nomogram in predicting the survival prognosis of patients with CRC within the TCGA cohort employing ROC curves that yielded AUC values of 0.676, 0.743, and 0.84 for predicting 1-, 3-, and 5-year survival, respectively (Figure 2H). The nomogram calibration plot further validates the robustness of our predictions, demonstrating substantial concordance between the nomogram-derived prognostications and the observed probabilities, as portrayed in Figure 2I–K.

### 3.6. Functional Analysis Based on the Platelet- and Coagulation-Related Risk Signature

**Figure 3:**
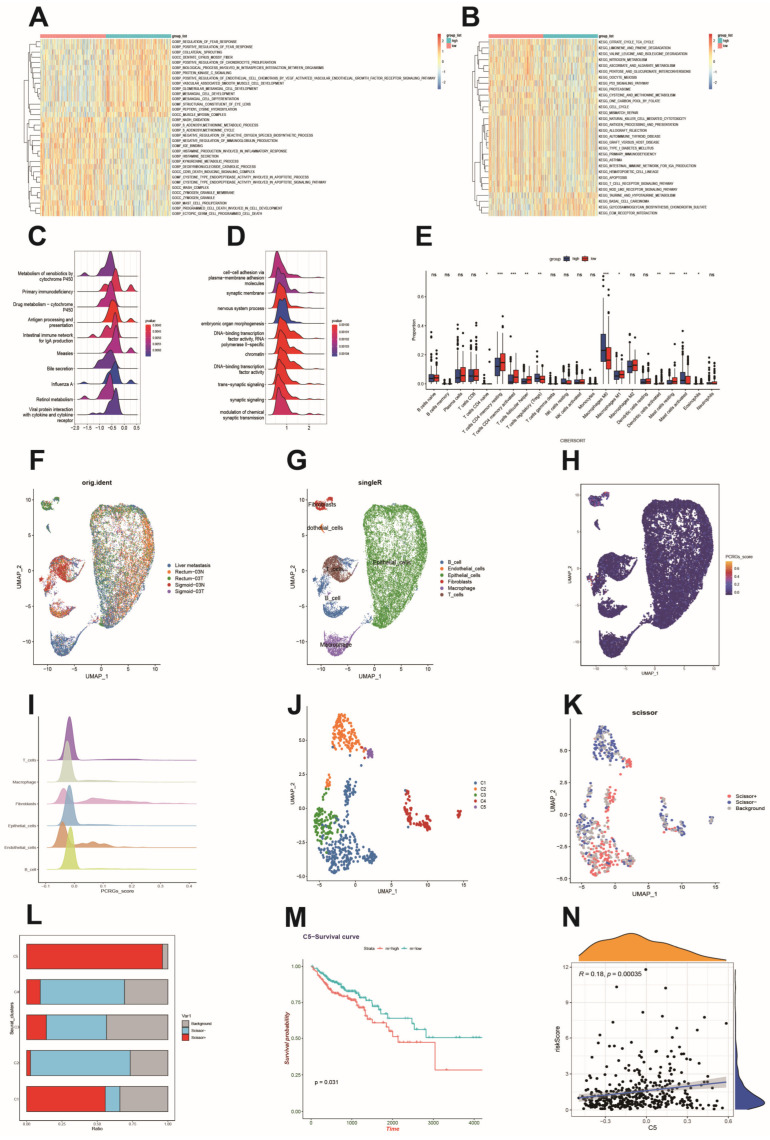
Functional enrichment analysis and scRNA-seq findings based on the CRC risk score. (**A**) GSVA-based GO enrichment analysis between risk groups. (**B**) GSVA-based KEGG enrichment analysis between risk groups. (**C**) Ridgeline plots depicting KEGG pathways enriched in each risk group according to GSEA. (**D**) Ridgeline plots depicting GO pathways enriched in each risk group according to GSEA. (**E**) Boxplots of differences in infiltrating immune cells between the risk groups. (**F**,**G**) UMAP plots of 21796 cells colored by sample or cell type. (**H**) Scatter plots showing the distribution of risk score expression in different cell clusters. (**I**) Ridgeline plots indicating the distribution of risk scores for different cell types. (**J**) UMAP plot depicting clustering of CRC-associated fibroblasts into 5 subtypes. (**K**) Metastasis-associated fibroblasts were identified using the Scissor package; Scissor+ cells (M1-stage-associated) are shown in red, and Scissor- cells (M0-stage-associated) in blue. (**L**) Comparative proportions of Scissor+ and Scissor- cells across different fibroblast subpopulations. (**M**) Survival analysis in the TCGA-COAD cohort, based on the C5 fibroblast subtype score. (**N**) Dot plot of the correlation between C5 fibroblast subtype score and model risk score in the TCGA-COAD cohort. * *p* < 0.05; ** *p* < 0.01; *** *p* < 0.001.

We further explored the biological functional differences between the high-risk and low-risk groups through GSVA and GSEA analyses. Our GSVA results, focusing on the GO section, revealed considerable enrichment in patients with high risk for terms such as “protein kinase C signaling”, “positive regulation of endothelial cell chemotaxis by the vascular endothelial growth factor receptor signaling pathway”, and “vascular-associated smooth muscle cell development” within BP, “platelet binding growth factor” and “dihydropyrimidinase activity” within MF, and “vascular-associated smooth muscle cell development” within CC (Figure 3A and Supplementary File S5). These findings suggest a close association between high-risk scores and processes involving cellular growth and tumor angiogenesis, two key aspects of tumorigenesis and cancer progression. Furthermore, our analysis unveiled several KEGG pathways, including “taurine and phospholipid metabolism”, “basal cell carcinoma”, “ECM receptor interactions”, and “focal adhesion”, with notable enrichment in the high-risk group (Figure 3B and Supplementary File S6). To validate the biological annotations attributed to the high-risk group, we conducted GSEA. This analysis revealed key GO pathways enriched in patients with high-risk “cell–cell adhesion via plasma membrane adhesion molecules”, “chromatin”, and “DNA-binding transcription factor activity” (Supplementary Figure S3A). The relative enrichment of the top 10 GO pathways was visually represented in a ridge diagram (Figure 3D). Meanwhile, in KEGG analysis, the top pathways associated with high-risk scores included “metabolism of xenobiotics by cytochrome P450”, “retinol metabolism”, and “primary immunodeficiency”, in addition to “primary metabolism” (Figure 3C and Supplementary Figure S3B).

### 3.7. Immune Cell Infiltration and Its Correlation with Hub Genes

To delve deeper into the distinctions in immune cell infiltration profiles between the high- and low-risk groups, we employed the CIBERSORT algorithm. The analysis revealed a significantly higher presence of eosinophils, naïve CD4 T-cells, regulatory T-cells (Tregs), M0 macrophages, and activated mast cells in the high-risk group compared to the low-risk group (Figure 3E). This observation suggests that these immune cell populations contribute to disease progression in the high-risk group. Furthermore, we computed correlation coefficients separately between risk PCRGs and immune cells, and explored the differential expression of immune checkpoint genes and HLA genes related to antigen presentation and immune response. Notably, there is a significant association between the mRNA levels of the 10 identified risk genes and the infiltration of various immune cell populations within the tumor microenvironment (Supplementary Figure S3C). Most of these genes exhibited elevated expression levels in the low-risk group, implying a more immunosuppressive TME in the high-risk group of patients (Supplementary Figure S3D,E).

### 3.8. Single-Cell Sequencing Reveals a Relationship between Risk Scores and the Tumor Microenvironment

To explore the pathobiological implications of the risk scores, we conducted an in-depth analysis using single-cell sequencing. We integrated and de-batched five samples from the GSE221575 dataset (Figure 3F) and categorized the cells into five distinct types: B-cells, T-cells, fibroblasts, macrophages, and epithelial cells (Figure 3G). To visualize risk score distribution across different cell clusters, we utilized the “AUC” package, along with UMAP and ridge plots. Notably, ten prognostic genes exhibited the highest expression levels in fibroblasts, implying a potential link between these genes and fibroblasts in the TME (Figure 3H,I). Based on AUC values, we further segregated all the fibroblasts. Subsequently, UMAP analysis enabled us to recluster these cells into five distinct subpopulations, denoted as C1 to C5, predicated on the similarity of gene expression patterns (Figure 3J). Furthermore, we used the “Scissor” package to analyze the association between fibroblasts from patients with CRC and clinical M-staging. The occurrence of scissor + cells was linked to metastasis, whereas scissor − cells were connected to non-metastasis. When these cellular attributes were mapped onto the fibroblast population, the highest frequency of scissor + cells showed the strongest association with the metastatic phenotype in the C5 fibroblast subtype. (Figure 3K,L). Subsequent survival analyses in the TCGA-COAD cohort based on C5 subtype characteristics substantiated that this specific fibroblast subpopulation was associated with poorer prognosis (Figure 3M). In turn, scatter plots further illustrated a positive correlation between C5 subgroup characterization scores and PCRGs risk scores in the TCGA-COAD cohort (Figure 3N).

### 3.9. High CYP19A1 Expression Predicts Poor Prognosis in CRC

**Figure 4:**
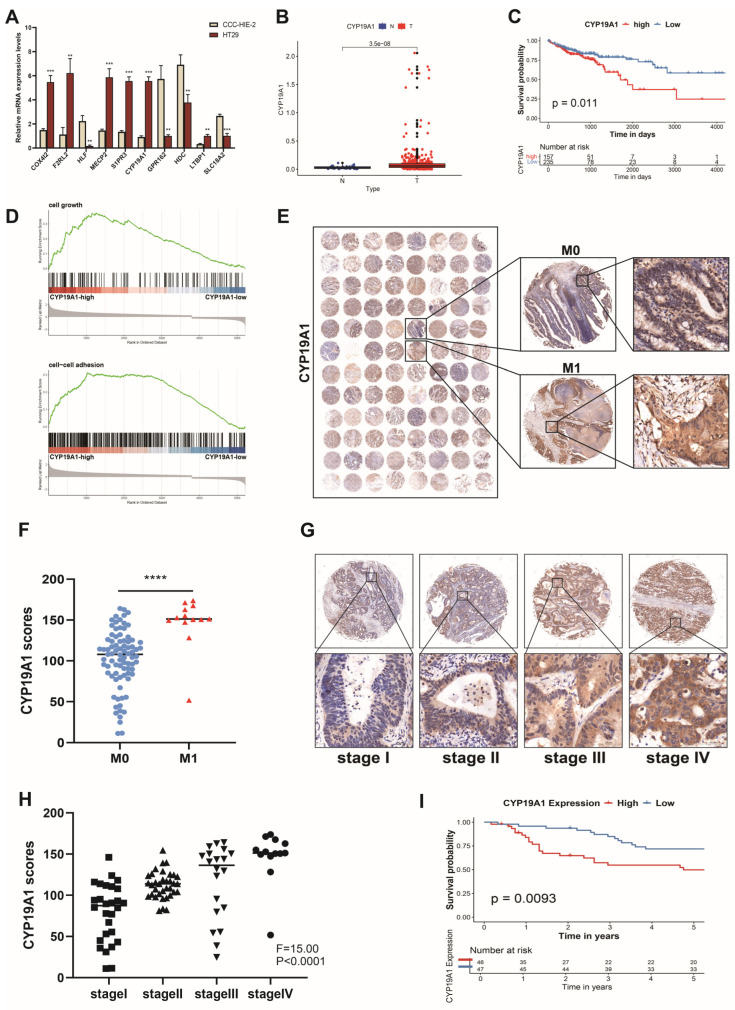
CYP19A1 upregulation correlates with poor prognosis in CRC. (**A**) Differential expression of 10 PCRGs in CRC HT29 cells and in normal colorectal CCC-HIE-2 cells. (**B**) Differential expression of the CYP19A1 gene between CRC and normal colorectal tissues in the TCGA-COAD cohort. (**C**) Kaplan–Meier (KM) curve for OS of patients in the TCGA-COAD dataset. Patients were stratified into low and high expression groups based on median CYP19A1 expression. (**D**) GSEA plots showing enrichment of genes related to cell growth and cell–cell adhesion in patients with high vs. low CYP19A1 expression in the TCGA-COAD dataset. (**E**,**F**) Immunohistochemical validation of CYP19A1 expression levels in M1 stage vs. M0 stage tumor tissues. Scale bar = 50 μm. (**G**,**H**) Immunohistochemical analysis of CYP19A1 expression across different pathological stages. Scale bar = 50 μm. (**I**) Prognostic analysis of CYP19A1 in 96 CRC patient samples in a tissue microarray. ** *p* < 0.01; *** *p* < 0.001; **** *p* < 0.0001. The black line represents the median value.

To investigate the 10 PCRGs included in our prognostic risk model, we first conducted an expression analysis in a CRC cell line (HT29) and a normal colorectal cell line (CCC-HIE-2) using RT-qPCR. Notably, CYP19A1 demonstrated pronounced upregulation in the former (Figure 4A). Furthermore, among the 10 PCRGs in the risk model, CYP19A1 exhibited the highest weight coefficient, prompting its selection for detailed investigation. Subsequent analysis of the TCGA-COAD dataset revealed significantly elevated expression of CYP19A1 in tumor tissues compared to adjacent non-tumor tissues (*p* < 0.05) (Figure 4B). Accessing TCGA datasets through the OncoLnc online tool, we next conducted survival analysis based on CYP19A1 expression in patients with COAD. We verified that patients with high CYP19A1 expression exhibited reduced survival (red line), contrasting with a more favorable survival outcome observed in patients with low CYP19A1 expression (*p* = 0.011) (Figure 4C).

To infer the role of CYP19A1 in CRC, we performed GSEA using TCGA-COAD RNA-seq data. GSEA plots highlighted a considerable enrichment of gene sets linked to cell growth and cell–cell adhesion in patients with elevated CYP19A1 expression (Figure 4D).

Subsequently, CYP19A1 immunohistochemistry was performed on a tissue microarray comprising 96 tumor samples from patients with CRC. The results indicated that CYP19A1 protein levels were elevated in M1-stage compared to M0-stage tumors (Figure 4E,F), and that high CYP19A1 expression may be associated with advanced pathological staging (Figure 4G,H). Additionally, CYP19A1 overexpression in the tissue microarray correlated with poor prognosis (Figure 4I). All this evidence prompted us to conduct further in vitro and in vivo functional assays to comprehensively explore the role of CYP19A1 in CRC.

### 3.10. CYP19A1 Promotes CRC Growth and Metastasis

**Figure 5:**
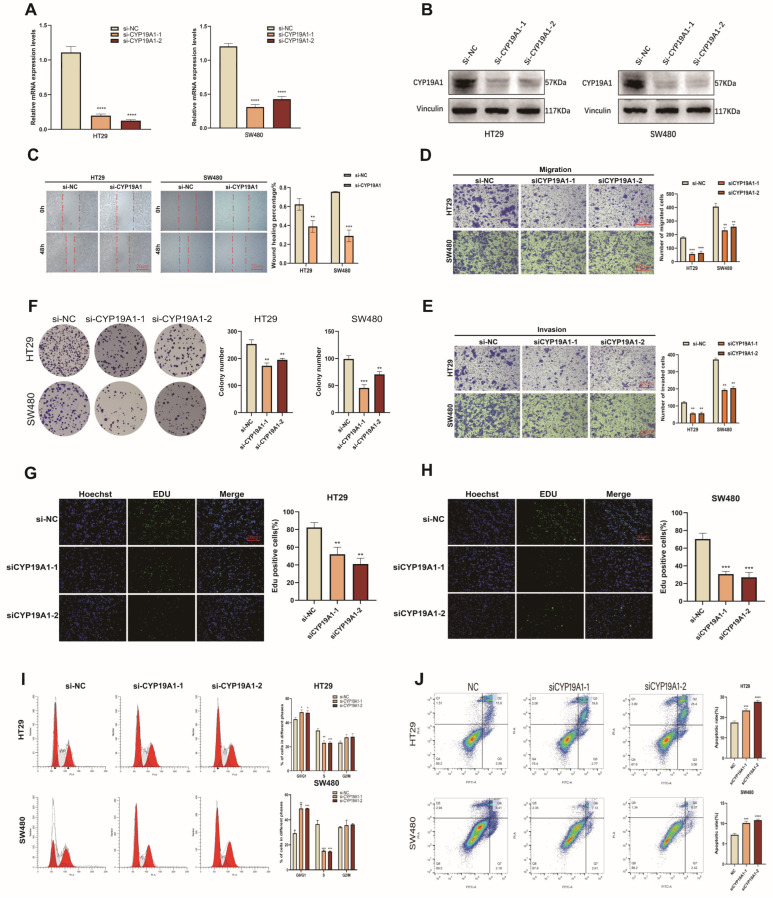
CYP19A1 silencing inhibits CRC cell migration, invasion, and proliferation. (**A**,**B**) Analysis of CYP19A1 mRNA (**A**) and protein (**B**) expression levels in HT29 and SW480 cells following transfection with two different CYP19A1 siRNA sequences, with si-NC serving as the negative control. (**C**) Representative images and quantification from wound-healing assays evaluating the impact of CYP19A1 silencing on CRC cell migration. (**D**,**E**) Representative images and quantification from Transwell assays assessing the effects of CYP19A1 silencing on the migration and invasion capabilities of CRC cells. (**F**) Representative images and quantification from colony formation assays to examine the influence of CYP19A1 silencing on the tumorigenic potential of HT29 and SW480 cells. (**G**,**H**) Representative images and quantification from EdU incorporation assays measuring proliferation in HT29 (**G**) and SW480 (**H**) cells. (**I**) Flow cytometric analysis of the cell cycle on the 7th day post-transfection with si-CYP19A1. (**J**) Flow cytometric analysis of apoptosis in CRC cells using Annexin V-FITC staining. * *p* < 0.05; ** *p* < 0.01; *** *p* < 0.001; **** *p* < 0.0001.

To assess the functional significance of CYP19A1 in CRC, we investigated alterations in cellular phenotypes following CYP19A1 knockdown in CRC cell lines. Efficient suppression of CYP19A1 expression was achieved in the HT29 and SW480 cell lines using two distinct small interfering RNAs (siRNAs) (Figure 5A,B). Subsequent wound healing and Transwell assays demonstrated that CYP19A1 knockdown significantly impairs the migratory and invasive capabilities of CRC cells. (Figure 5C–E). These findings suggest that CYP19A1 plays a key role in promoting invasion and migration in CRC cells. Furthermore, colony formation and 5-ethynyl-2’-deoxyuridine (EdU) incorporation assays confirmed that CYP19A1 significantly enhances the proliferative capacity of CRC cells, as demonstrated by a marked reduction in colony numbers (Figure 5F) and a decrease in EdU incorporation into replicating DNA (Figure 5G,H). Complementing these findings, cell cycle analysis revealed that CYP19A1 deficiency led to pronounced G0/G1 arrest (Figure 5I). Moreover, apoptosis assays underscored an enhanced apoptotic response in CYP19A1-deficient CRC cells compared to those transfected with siRNA-negative control (si-NC) (Figure 5J).

**Figure 6:**
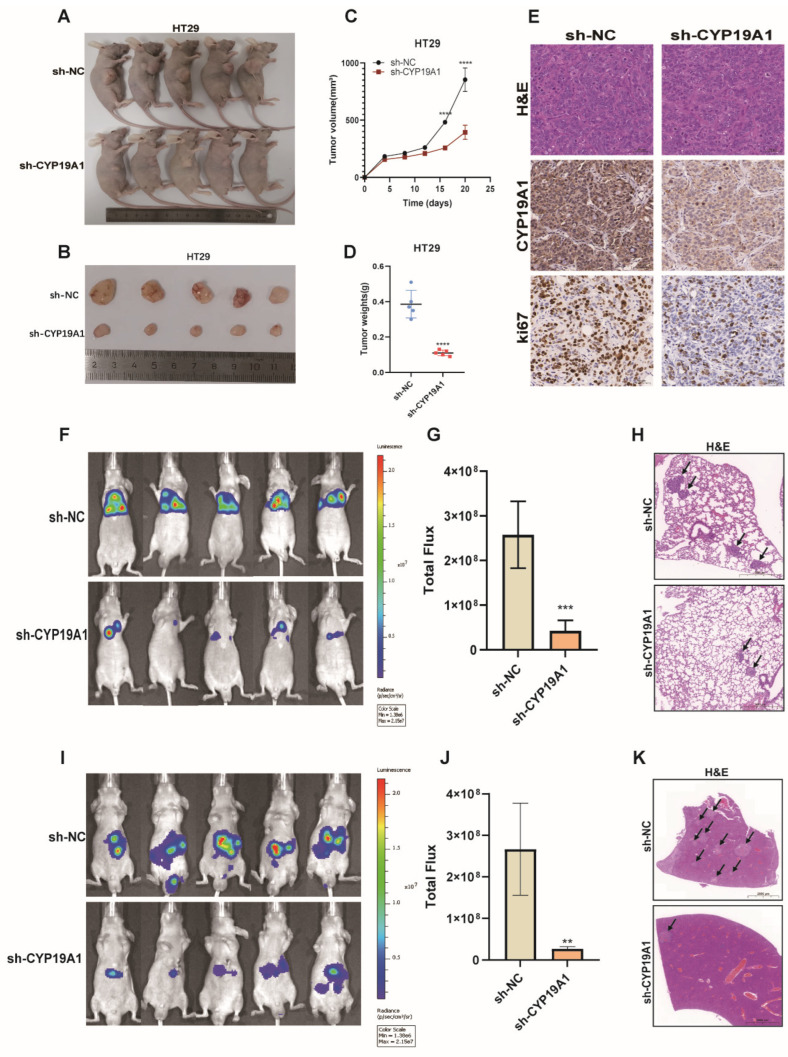
CYP19A1 silencing inhibits proliferative and metastatic behavior of CRC cells in vivo. Xenografts were established in nude mice by inoculating HT29 cells transfected with either sh-NC or sh-CYP19A1. (**A**,**B**) Representative photographs of HT29 cell xenografts in situ (**A**) and excised tumors (**B**). (**C**) Tumor volume growth curve. Data are shown as mean ± SEM of five mice in each group (two-way ANOVA). (**D**) Excised tumor weight (Mann–Whitney test). (**E**) Representative H&E staining and CYP19A1 and Ki-67 immunohistochemistry images from HT29 cell xenografts. Statistical analyses were carried out using Student’s *t*-test. (**F**,**G**) Bioluminescence images of mice intravenously injected with HT29 cells transfected with either sh-NC or sh-CYP19A1 and corresponding quantification of bioluminescence intensities (*n* = 5). (**H**) Representative H&E staining images of lung tissues showing metastatic lung foci (arrows) in mice intravenously injected with HT29 cells. (**I**,**J**) Bioluminescence imaging of mouse livers and corresponding quantification of bioluminescence intensity (*n* = 5). (**K**) Representative H&E-stained images of mouse liver tissue displaying metastatic liver lesions (arrow). ** *p* < 0.01; *** *p* < 0.001; **** *p* < 0.0001.

To confirm the oncogenic role of CYP19A1 in vivo, we developed a subcutaneous xenograft tumor model in nude mice and observed that CYP19A1 knockdown markedly suppressed subcutaneous tumor growth (Figure 6A–C). At the conclusion of the animal study, tumors were harvested and weighed. Tumor weight in the CYP19A1 knockdown group was significantly reduced compared to the control group (Figure 6D). Accordingly, Ki-67 staining indicated a significantly decreased proliferative capacity for CYP19A1-deficient cells within the xenografts (Figure 6E).

To establish metastatic lung and liver colonization models, HT29 cells co-expressing a luciferase reporter plasmid were injected into the tail vein and spleen of BALB/c nude mice, respectively. The progression of lung and liver lesions was monitored using bioluminescence imaging. After 5 weeks, the mice were sacrificed, and lung and liver tissues were subjected to H&E staining. In vivo imaging demonstrated that CYP19A1 silencing attenuated lung metastasis (Figure 6F,G) and liver metastasis (Figure 6I,J). Representative H&E-stained images of mouse lung tissue displayed metastatic lung lesions (arrows) (Figure 6H), while representative images of mouse liver tissue revealed metastatic liver lesions (arrows) (Figure 6K). Knockdown of CYP19A1 markedly decreases the incidence of both pulmonary and hepatic metastases. These findings strongly suggest that CYP19A1 promotes the proliferation and metastasis of CRC cell lines.

### 3.11. CYP19A1 Expression in CRC Cells Activates on Platelet Activation In Vitro

**Figure 7:**
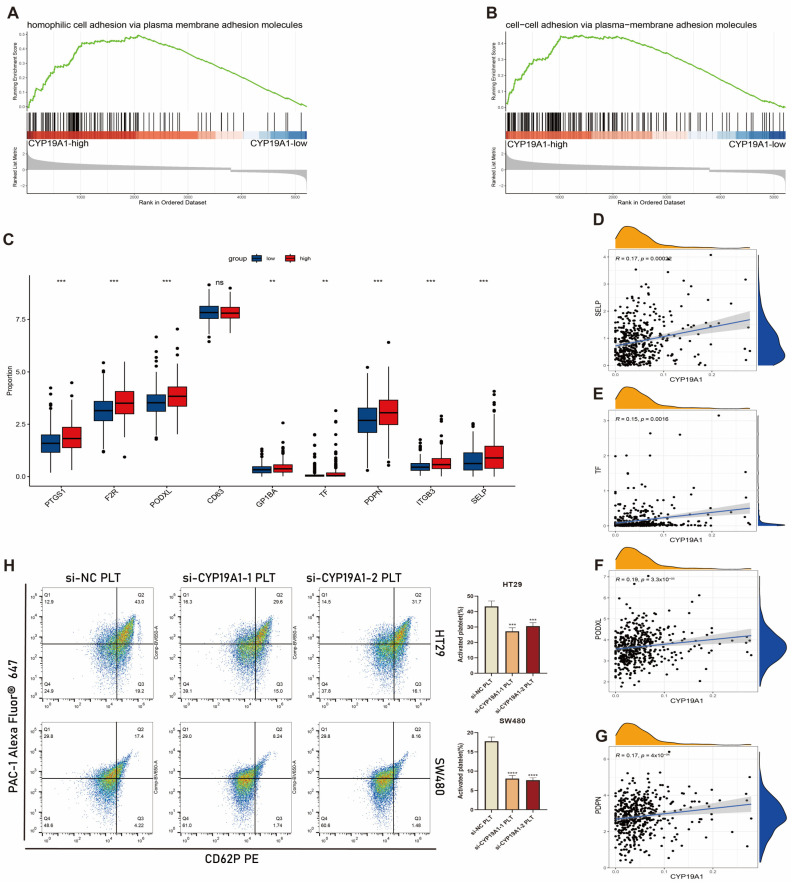
CYP19A1 expression in CRC cells promotes platelet activation. (**A**,**B**) GSEA plots illustrating the enrichment of genes associated with “homophilic cell adhesion via plasma membrane adhesion molecules” and “cell–cell adhesion via plasma membrane adhesion molecules” in patients with high versus low CYP19A1 expression within the TCGA COAD dataset. (**C**) Boxplots depicting differences in the expression of platelet activation-related genes among high and low CYP19A1 expression groups. (**D**–**G**) Scatter plots showing the correlation between CYP19A1 and SELP, TF, PODXL, and PDPN genes. (**H**) Flow cytometry analysis of platelets co-cultured with CRC cells. HT29 and SW480 cells were transfected with si-NC, si-CYP19A1, or si-CYP19A2 and then co-cultured with platelets (PLT). Platelet activation was quantified based on CD62P and PAC-1 surface expression. ns *p* > 0.05; ** *p* < 0.01; *** *p* < 0.001; **** *p* < 0.0001.

After substantiating the involvement of CYP19A1 in CRC progression through the above in vitro and in vivo analyses, we asked whether intratumoral CYP19A1 expression levels influence platelet activation with consequent modulation of the coagulation cascade. Using information from the TCGA-COAD dataset, GSEA revealed a pronounced enrichment in gene sets linked to “homophilic cell adhesion via plasma membrane adhesion molecules” (Figure 7A) and “cell–cell adhesion via plasma membrane adhesion molecules” (Figure 7B) in patients exhibiting elevated CYP19A1 expression. Contrarily, such enrichment was essentially absent in patients characterized by low expression of CYP19A1. Interactions via homophilic binding mediated by plasma membrane adhesion molecules are integral to the orchestration of platelet activation and aggregation, thereby considerably contributing to the blood coagulation cascade [34].

We next reinterrogated the TCGA database to assess the expression profiles of nine genes implicated in platelet activation and aggregation, comparing cohorts with high versus low expression of CYP19A1. Our analysis revealed a notable upregulation of eight genes (with the exception of CD63) in the high CYP19A1 expression group (Figure 7C). Notably, CYP19A1 demonstrated a robust positive correlation with key genes involved in platelet activation, including SELP, TF, PODXL, and PDPN [35,36,37,38] (Figure 7D–G).

Prompted by these observations, we postulated that CYP19A1 expression in CRC cells might be a determinant of platelet activation. To test this hypothesis in an in vitro setting, we performed siRNA-mediated silencing of CYP19A1 in CRC cells, and utilized them to directly stimulate isolated platelets. Results of flow cytometric analysis conducted to quantify the extent of platelet activation demonstrated that CYP19A1 knockdown in CRC cells led to marked inhibition of platelet activation, relative to cells transfected with negative control siRNA (Figure 7H).

These findings preliminarily indicate that CYP19A1 plays a dual role, i.e., modulating malignant cellular behaviors in CRC and concurrently influencing platelet activation. This dual functionality positions CYP19A1 as a pivotal molecular intermediary in the interplay between CRC cells and platelets, potentially contributing to the pathogenesis of CRC.

## 4. Discussion

Disruptions in platelet and coagulation functions are closely linked to malignant tumor progression, offering new insights into cancer treatment. However, the precise roles of platelets and the coagulation pathway in various tumor types remain poorly understood, and their relationship with CRC is still unclear.

In this study, we systematically gathered a comprehensive panel of 376 PCRGs through the integration of WGCNA and data culled from diverse databases. From this expansive gene set, we developed a prognostic model consisting of ten genes by employing Lasso Cox analysis. Six of them, namely LTBP1, COX4I2, CYP19A1, F2RL2, MECP2, and S1PR3, demonstrated notable upregulation in CRC samples, while the remaining four genes, HLF, HDC, SLC18A2, and GPR162, exhibited comparatively lower expression. Previous research endeavors have underlined the close association of these genes with tumor initiation, progression, and the TME. For instance, a study by Zhao et al. unveiled the significance of PTPS, which exhibits heightened expression in early colon cancer. Under hypoxic conditions, AMPK-mediated phosphorylation of PTPS at Thr 58 prompts its binding to LTBP1, fostering iNOS-mediated LTBP1 S-nitrosylation within the PTPS/iNOS/LTBP1 complex, leading to proteasome-dependent LTBP1 degradation. Functionally, the inhibition of LTBP1 by PTPS leads to impaired secretion of TGF-β, thereby sustaining tumor cell growth under hypoxic conditions [39]. CYP19A1-catalyzed estrogen biosynthesis has been implicated in vascular abnormalities and suppression of CD8+ T-cell function through the upregulation of PD-L1, IL-6, and TGF-β via the GPR30-AKT signaling pathway. In the context of colon cancer immunotherapy, the inhibition of CYP19A1 combined with PD-1 blockade was proposed as a promising therapeutic strategy [40]. Notably, Zhang et al. identified a notable association between germline deletions affecting the last six exons of SLC18A1 and CRC [41]. Histidine decarboxylase (HDC) deficiency has been shown to promote inflammation-associated CRC by provoking the accumulation of CD11b+Gr-1+ immature myeloid cells [42]. Meanwhile, reports suggest that targeting S1PR3 may enhance the antitumor efficacy of CAR T-cell therapy by inhibiting T-cell exhaustion and reshaping the TME through the recruitment of pro-inflammatory macrophages [43]. Elevated levels of COX4I2 have been linked to unfavorable clinical prognosis. Additionally, COX4I2 may be associated with fibroblast growth factor 1 (FGF-1), which plays pivotal roles in EMT, cancer-associated fibroblast activation, and angiogenesis [44]. Lastly, MeCP2 has been shown to bind to methyltransferase-like 14 (METTL14), thereby promoting CRC metastasis and progression through the modulation of the tumor suppressor Krüppel-like factor 4 (KLF4) via m6A methylation core modification [45]. As the role of the remaining risk genes, i.e., HLF, GPR162, and F2RL2, in CRC remains relatively unexplored, further investigations are warranted to assess their relevance.

Following the construction of a platelet-coagulation-related risk profile, we conducted a series of comprehensive bioinformatic analyses to ascertain its significance in CRC. Following stratification of patients with CRC into distinct risk groups based on median risk scores, patients with high risk exhibited a considerably poorer prognosis compared to their low-risk counterparts. These observations were further substantiated through validation in two independent CRC cohorts, and ROC curves corroborated the robustness and precision of our prognostic risk model. Furthermore, our enrichment analyses and investigations into immune infiltration (comprising GSEA, GSVA, and CIBERSORT) unveiled compelling disparities in biological functions and composition of the immune microenvironment among individuals from different risk groups. This elucidation holds promise for the identification of intervention targets and the development of individualized treatment strategies. In addition, leveraging single-cell sequencing analysis, we identified ten prognostic genes that exhibited substantial enrichment in fibroblasts. Subsequently, fibroblasts were subjected to re-clustering into five distinct subpopulations. Employing the “Scissor” software package, we meticulously examined fibroblasts from patients with CRC to identify the fibroblast subpopulation most closely linked to metastasis. Intriguingly, this subpopulation not only had notable implications for patient survival but also displayed a positive correlation with risk scores embedded within our prognostic models.

We subsequently focused on cytochrome P450 family 19 subfamily A member 1 (CYP19A1) to conduct detailed biological function experiments. CYP19A1 encodes the enzyme aromatase, which is crucial for converting androgens into estrogens [46]. This enzyme plays a significant role in various physiological processes, including the regulation of estrogen levels in tissues [46]. In cancer, particularly in hormone-dependent tumors such as breast and ovarian cancers, CYP19A1 is implicated in tumor progression through its role in estrogen biosynthesis, positioning it as a potential biomarker [47]. Our in vitro and in vivo studies demonstrate that CYP19A1 promotes malignant biological behaviors, including tumor proliferation, invasion, and migration, as well as platelet activation in vitro. This dual functionality may be crucial in the malignant progression of colorectal tumors.

In summary, our investigation presents, for the first time, compelling evidence of a substantial correlation between platelet-coagulation-related genes and the prognosis of patients with CRC. We have successfully developed an autonomous prognostic model, centered on genes related to the platelet-coagulation system, which serves as a robust predictor of CRC prognosis. The model’s autonomy and predictive efficacy were subsequently confirmed through rigorous validation against external datasets. Our research increases our understanding of platelets’ relevance in the context of CRC, offering novel and promising avenues for prognostic assessment as well as potential therapeutic biomarkers.

## Data Availability

The datasets generated and/or analyzed during the current study are available in the TCGA repository, https://portal.gdc.cancer.gov/ (accessed on 22 May 2024), and GEO repository, https://www.ncbi.nlm.nih.gov/geo/ (accessed on 22 May 2024).

## Supplementary Materials

The following supporting information can be downloaded at: https://www.mdpi.com/article/10.3390/biomedicines12102225/s1, Figure S1: (A) Determination of soft-thresholding power in the WGCNA. (B) The cluster dendrogram of the genes with median absolute deviation in the top 25%. Each branch in the figure represents one gene, and every color below represents one co-expression module. (C) Explore correlations between modules that have low coefficients in all regions except for the high diagonal coefficient. (D) GO enrichment analysis of platelet-related gene sets. (E) KEGG enrichment analysis of platelet-related gene sets. (F) The expression pattern of model genes in the TCGA-CRC cohort; Figure S2: (A–F) The risk score delamination, survival state, as well as the expression pattern of model genes in the GSE17536 (left) and GSE39582 cohorts (right); Figure S3: (A) GO pathways enriched in each of the two risk groups according to the GSEA methodology and visualized with ridge diagrams. (B) KEGG pathways enriched in each of the two risk groups according to the GSEA approach, and visualized with ridge maps. (C) Correlation matrix between model genes and infiltrating immunocytes. (D,E) Boxplots of the discrepancies in the expression of immune checkpoint and human leukocyte antigen (HLA) genes between distinct groups (* *p* < 0.05; ** *p* < 0.01; *** *p* < 0.001); Table S1: Primer sequence list .

## Abbreviations

CRC: colorectal carcinoma; PCRG, platelet- and coagulation-related gene; GEO, gene expression omnibus; GO, gene ontology; GSEA, gene set enrichment analysis; KEGG, Kyoto Encyclopedia of Genes and Genomes; KM, Kaplan–Meier; OS, overall survival; ROC, receiver operating characteristic; TCGA, The Cancer Genome Atlas; TME, tumor microenvironment; WGCNA, weighted gene co-expression network analysis; GSVA, gene set variation analysis.

## References

[1] Jemal A., Bray F., Center M.M., Ferlay J., Ward E., Forman D. (2011). Global cancer statistics. CA Cancer J. Clin..

[2] Bray F., Ferlay J., Soerjomataram I., Siegel R.L., Torre L.A., Jemal A. (2018). Global cancer statistics 2018: GLOBOCAN estimates of incidence and mortality worldwide for 36 cancers in 185 countries. CA Cancer J. Clin..

[3] Siegel R.L., Wagle N.S., Cercek A., Smith R.A., Jemal A. (2023). Colorectal cancer statistics, 2023. CA Cancer J. Clin..

[4] Gaedcke J., Grade M., Camps J., Søkilde R., Kaczkowski B., Schetter A.J., Difilippantonio M.J., Harris C.C., Ghadimi B.M., Møller S. (2012). The rectal cancer microRNAome—microRNA expression in rectal cancer and matched normal mucosa. Clin. Cancer Res..

[5] Siegel R.L., Miller K.D., Jemal A. (2019). Cancer statistics, 2019. CA Cancer J. Clin..

[6] Luo Y., Cui J., Chen C., Song S., Huang M., Peng J., Lan P., Huang Y., Wang L., Wang J. (2012). Clinical outcomes after surgical resection of colorectal cancer in 1294 patients. Hepatogastroenterology.

[7] Delaunoit T., Limburg P.J., Goldberg R.M., Lymp J.F., Loftus E.V. (2006). Colorectal cancer prognosis among patients with inflammatory bowel disease. Clin. Gastroenterol. Hepatol..

[8] Dogan L., Karaman N., Yilmaz K.B., Ozaslan C., Atalay C., Altinok M. (2010). Characteristics and risk factors for colorectal cancer recurrence. J. BUON.

[9] Donati M.B. (1995). Cancer and thrombosis: From Phlegmasia alba dolens to transgenic mice. Thromb. Haemost..

[10] Sylman J.L., Mitrugno A., Atallah M., Tormoen G.W., Shatzel J.J., Yunga S.T., Wagner T.H., Leppert J.T., Mallick P., McCarty O.J.T. (2018). The Predictive Value of Inflammation-Related Peripheral Blood Measurements in Cancer Staging and Prognosis. Front. Oncol..

[11] Joosse S.A., Pantel K. (2015). Tumor-Educated Platelets as Liquid Biopsy in Cancer Patients. Cancer Cell.

[12] Best M.G., Wesseling P., Wurdinger T. (2018). Tumor-Educated Platelets as a Noninvasive Biomarker Source for Cancer Detection and Progression Monitoring. Cancer Res..

[13] Labelle M., Begum S., Hynes R.O. (2011). Direct signaling between platelets and cancer cells induces an epithelial-mesenchymal-like transition and promotes metastasis. Cancer Cell.

[14] Klinger M.H., Jelkmann W. (2002). Role of blood platelets in infection and inflammation. J. Interferon Cytokine Res..

[15] Haemmerle M., Stone R.L., Menter D.G., Afshar-Kharghan V., Sood A.K. (2018). The Platelet Lifeline to Cancer: Challenges and Opportunities. Cancer Cell.

[16] In’t Veld S., Wurdinger T. (2019). Tumor-educated platelets. Blood.

[17] Sylman J.L., Mitrugno A., Tormoen G.W., Wagner T.H., Mallick P., McCarty O.J.T. (2017). Platelet count as a predictor of metastasis and venous thromboembolism in patients with cancer. Converg. Sci. Phys. Oncol..

[18] Gardiner C., Harrison P., Belting M., Böing A., Campello E., Carter B.S., Collier M.E., Coumans F., Ettelaie C., van Es N. (2015). Extracellular vesicles, tissue factor, cancer and thrombosis—Discussion themes of the ISEV 2014 Educational Day. J. Extracell. Vesicles.

[19] Bastida E., Ordinas A., Escolar G., Jamieson G.A. (1984). Tissue factor in microvesicles shed from U87MG human glioblastoma cells induces coagulation, platelet aggregation, and thrombogenesis. Blood.

[20] Khorana A.A., Francis C.W., Culakova E., Kuderer N.M., Lyman G.H. (2007). Thromboembolism is a leading cause of death in cancer patients receiving outpatient chemotherapy. J. Thromb. Haemost..

[21] Sallah S., Wan J.Y., Nguyen N.P. (2002). Venous thrombosis in patients with solid tumors: Determination of frequency and characteristics. Thromb. Haemost..

[22] Khorana A.A., Francis C.W., Culakova E., Lyman G.H. (2005). Risk factors for chemotherapy-associated venous thromboembolism in a prospective observational study. Cancer.

[23] Weinstein J.N., Collisson E.A., Mills G.B., Shaw K.R., Ozenberger B.A., Ellrott K., Shmulevich I., Sander C., Stuart J.M. (2013). The Cancer Genome Atlas Pan-Cancer analysis project. Nat. Genet..

[24] Barrett T., Troup D.B., Wilhite S.E., Ledoux P., Rudnev D., Evangelista C., Kim I.F., Soboleva A., Tomashevsky M., Edgar R. (2007). NCBI GEO: Mining tens of millions of expression profiles—Database and tools update. Nucleic Acids Res..

[25] Yue T., Chen S., Zhu J., Guo S., Huang Z., Wang P., Zuo S., Liu Y. (2021). The aging-related risk signature in colorectal cancer. Aging.

[26] Safran M., Dalah I., Alexander J., Rosen N., Stein T.I., Shmoish M., Nativ N., Bahir I., Doniger T., Krug H. (2010). GeneCards Version 3: The human gene integrator. Database.

[27] Bindea G., Mlecnik B., Tosolini M., Kirilovsky A., Waldner M., Obenauf A.C., Angell H., Fredriksen T., Lafontaine L., Berger A. (2013). Spatiotemporal dynamics of intratumoral immune cells reveal the immune landscape in human cancer. Immunity.

[28] Hänzelmann S., Castelo R., Guinney J. (2013). GSVA: Gene set variation analysis for microarray and RNA-seq data. BMC Bioinform..

[29] Newman A.M., Liu C.L., Green M.R., Gentles A.J., Feng W., Xu Y., Hoang C.D., Diehn M., Alizadeh A.A. (2015). Robust enumeration of cell subsets from tissue expression profiles. Nat. Methods.

[30] Stuart T., Butler A., Hoffman P., Hafemeister C., Papalexi E., Mauck W.M., Hao Y., Stoeckius M., Smibert P., Satija R. (2019). Comprehensive Integration of Single-Cell Data. Cell.

[31] Tosches M.A., Yamawaki T.M., Naumann R.K., Jacobi A.A., Tushev G., Laurent G. (2018). Evolution of pallium, hippocampus, and cortical cell types revealed by single-cell transcriptomics in reptiles. Science.

[32] Han X., Zhou Z., Fei L., Sun H., Wang R., Chen Y., Chen H., Wang J., Tang H., Ge W. (2020). Construction of a human cell landscape at single-cell level. Nature.

[33] Chi M., Liu J., Mei C., Shi Y., Liu N., Jiang X., Liu C., Xue N., Hong H., Xie J. (2022). TEAD4 functions as a prognostic biomarker and triggers EMT via PI3K/AKT pathway in bladder cancer. J. Exp. Clin. Cancer Res..

[34] Kunicki T.J. (1989). Platelet membrane glycoproteins and their function: An overview. Blut.

[35] Joseph J.E., Harrison P., Mackie I.J., Machin S.J. (1998). Platelet activation markers and the primary antiphospholipid syndrome (PAPS). Lupus.

[36] Gergei I., Kälsch T., März W., Krämer B.K., Kälsch A.I. (2020). Platelet and Monocyte Activity Markers and Mortality in Patients with End-Stage Renal Disease. Clin. Lab..

[37] Pericacho M., Alonso-Martín S., Larrucea S., González-Manchón C., Fernández D., Sánchez I., Ayuso M.S., Parrilla R. (2011). Diminished thrombogenic responses by deletion of the Podocalyxin Gene in mouse megakaryocytes. PLoS ONE.

[38] Wang X., Liu B., Xu M., Jiang Y., Zhou J., Yang J., Gu H., Ruan C., Wu J., Zhao Y. (2021). Blocking podoplanin inhibits platelet activation and decreases cancer-associated venous thrombosis. Thromb. Res..

[39] Zhao Q., Zheng K., Ma C., Li J., Zhuo L., Huang W., Chen T., Jiang Y. (2020). PTPS Facilitates Compartmentalized LTBP1 S-Nitrosylation and Promotes Tumor Growth under Hypoxia. Mol. Cell.

[40] Liu L., Mo M., Chen X., Chao D., Zhang Y., Chen X., Wang Y., Zhang N., He N., Yuan X. (2023). Targeting inhibition of prognosis-related lipid metabolism genes including CYP19A1 enhances immunotherapeutic response in colon cancer. J. Exp. Clin. Cancer Res..

[41] Zhang D., Li Z., Xu X., Zhou D., Tang S., Yin X., Xu F., Li H., Zhou Y., Zhu T. (2017). Deletions at SLC18A1 increased the risk of CRC and lower SLC18A1 expression associated with poor CRC outcome. Carcinogenesis.

[42] Yang X.D., Ai W., Asfaha S., Bhagat G., Friedman R.A., Jin G., Park H., Shykind B., Diacovo T.G., Falus A. (2011). Histamine deficiency promotes inflammation-associated carcinogenesis through reduced myeloid maturation and accumulation of CD11b+Ly6G+ immature myeloid cells. Nat. Med..

[43] Gao G., Liao W., Shu P., Ma Q., He X., Zhang B., Qin D., Wang Y. (2023). Targeting sphingosine 1-phosphate receptor 3 inhibits T-cell exhaustion and regulates recruitment of proinflammatory macrophages to improve antitumor efficacy of CAR-T cells against solid tumor. J. Immunother. Cancer.

[44] Li J.P., Liu Y.J., Zeng S.H., Gao H.J., Chen Y.G., Zou X. (2022). Identification of COX4I2 as a hypoxia-associated gene acting through FGF1 to promote EMT and angiogenesis in CRC. Cell Mol. Biol. Lett..

[45] Wang S., Gan M., Chen C., Zhang Y., Kong J., Zhang H., Lai M. (2021). Methyl CpG binding protein 2 promotes colorectal cancer metastasis by regulating N^6^-methyladenosine methylation through methyltransferase-like 14. Cancer Sci..

[46] Nelson L.R., Bulun S.E. (2001). Estrogen production and action. J. Am. Acad. Dermatol..

[47] Kharb R., Haider K., Neha K., Yar M.S. (2020). Aromatase inhibitors: Role in postmenopausal breast cancer. Arch. Pharm..

