# Supplementary material for: Comprehensive Analysis of a Platelet- and Coagulation-Related Prognostic Gene Signature Identifies CYP19A1 as a Key Tumorigenic Driver of Colorectal Cancer
Source: Biomedicines. 2024 Sep 30;12(10):2225. doi: 10.3390/biomedicines12102225 (PMC11505370; doi:10.3390/biomedicines12102225)
Supplement: Supplementary file 1 [file biomedicines-12-02225-s001.zip › Supplementary Figure.pdf]

Supplementary Figure

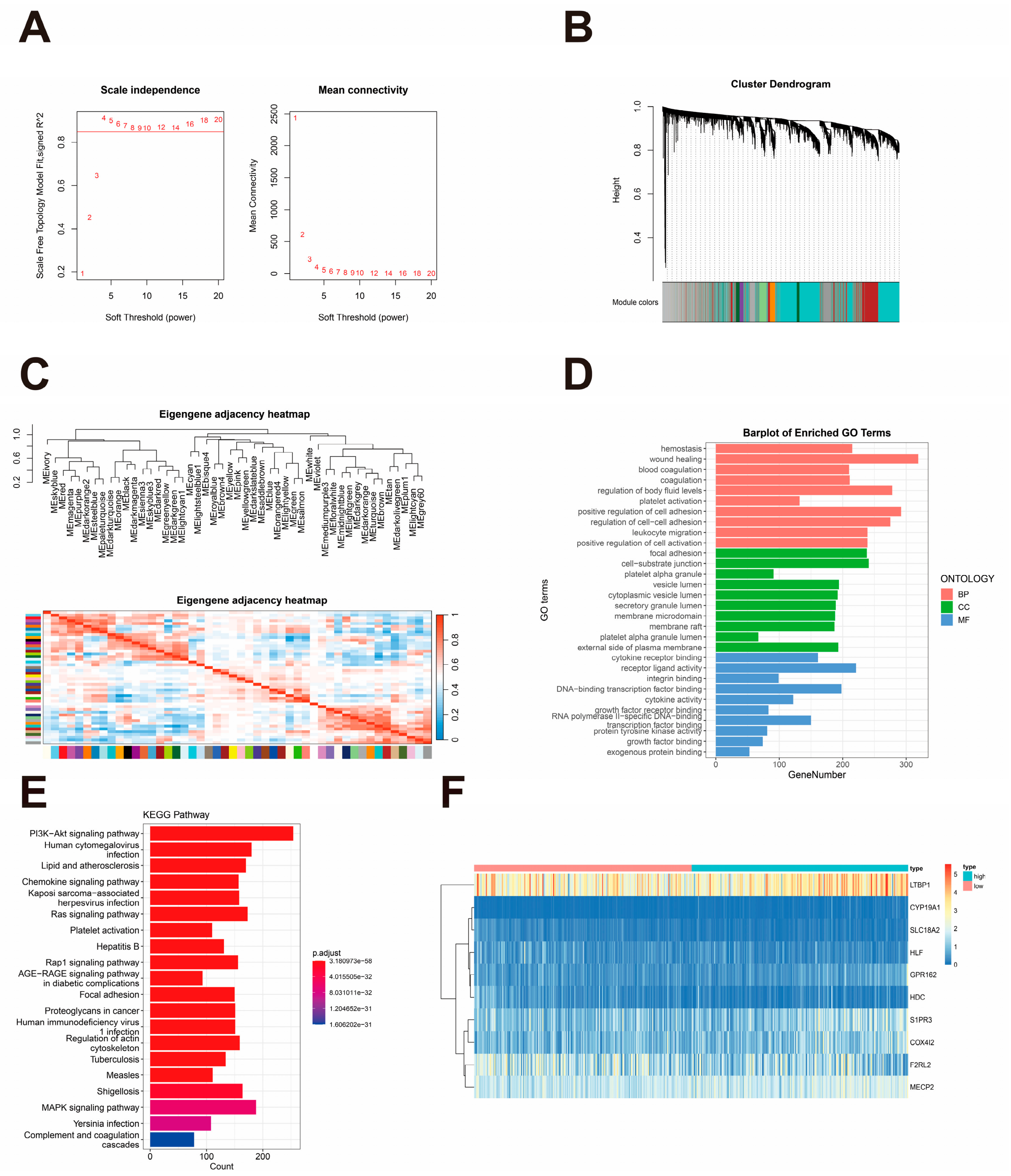

Supplementary Figure S1 (A) Determination of soft-thresholding power in the WGCNA.(B) The cluster dendrogram of the genes with median absolute deviation in the top 25%. Each branch in the figure represents one gene, and every color below represents one co-expression module.(C) Explore correlations between modules that have low coefficients in

all regions except for the high diagonal coefficient. (D) GO enrichment analysis of platelet-related gene sets.(E) KEGG enrichment analysis of platelet-related gene sets. (F) The expression pattern of model genes in the TCGA-CRC cohort.

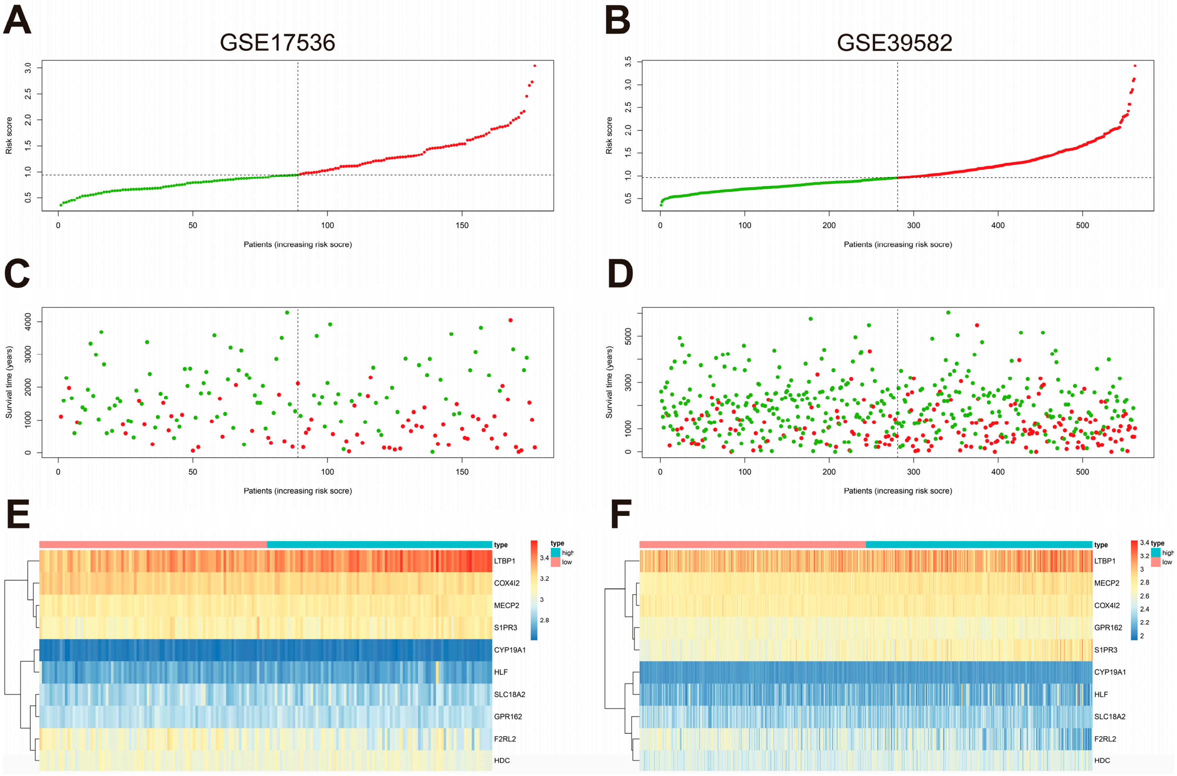

Supplementary Figure S2 (A-F) The risk score delamination, survival state, as well as the expression pattern of model genes in the GSE17536 (left) and GSE39582 cohorts (right).

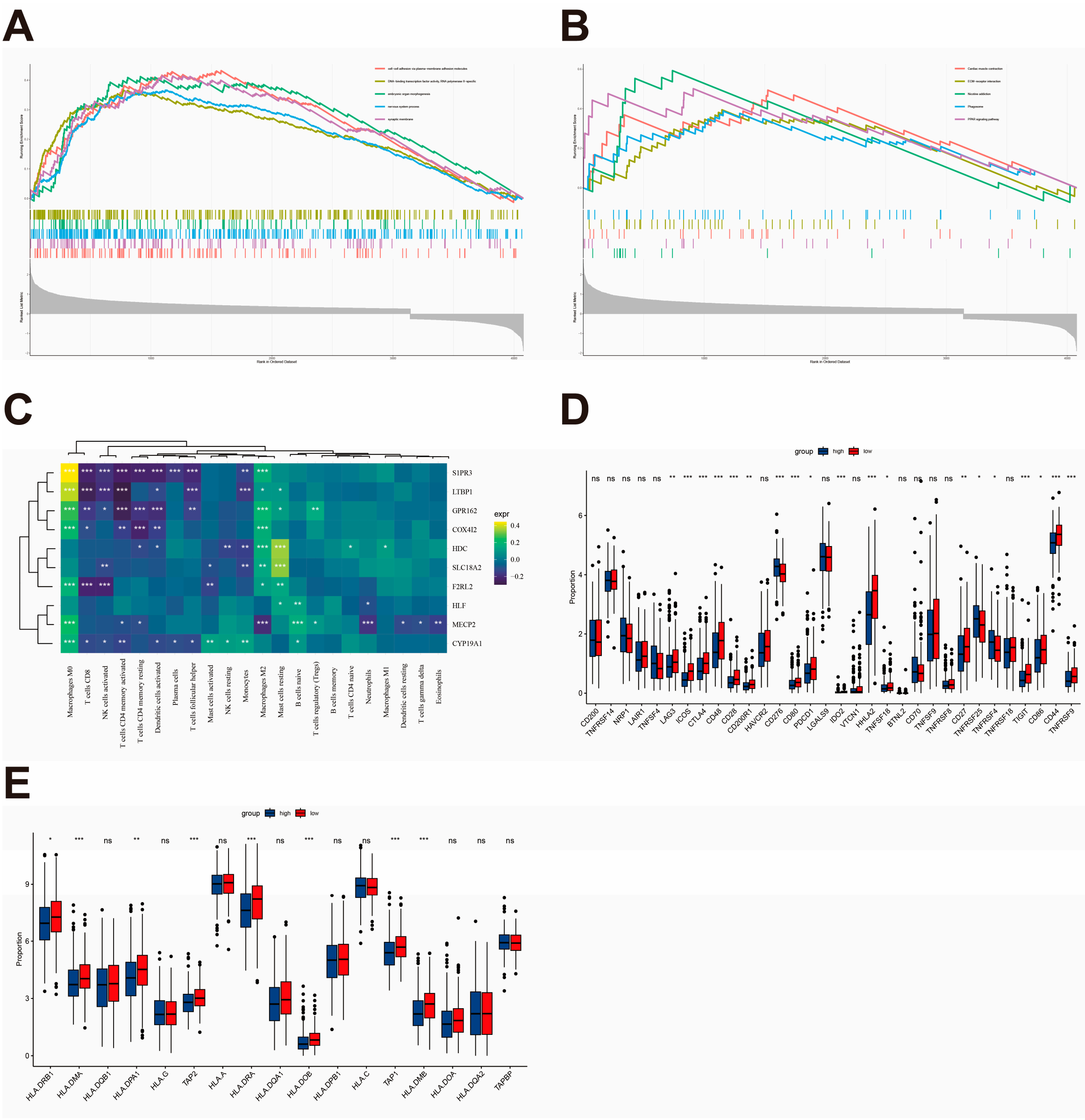

Supplementary Figure S3 (A) GO pathways enriched in each of the two risk groups according to the GSEA methodology and visualized with ridge diagrams. (B) KEGG pathways enriched in each of the two risk groups according to the GSEA approach,and visualized with ridge maps. (C)Correlation matrix between model genes and infiltrating immunocytes. (D-E) Boxplots of the discrepancies in the expression of immune checkpoint and human leukocyte antigen (HLA) genes between distinct groups(*p < 0.05; **p < 0.01; ***p < 0.001).
